# Secreted frizzled-related protein 4 (sFRP4) in cancer—Dual roles in tumorigenesis and therapeutic potential: A review

**DOI:** 10.17305/bb.2025.13047

**Published:** 2025-09-02

**Authors:** Yu Jiang, Luyao Wang, Yerong Li, Juan Liu, Juan Lv, Pengfei Xu

**Affiliations:** 1 Department of Gynecology, Women’s Hospital of Nanjing Medical University, Nanjing, China; 2 Nanjing Women and Children’s Healthcare Institute, Women’s Hospital of Nanjing Medical University, Nanjing, China

**Keywords:** sFRP4, tumorigenesis, dual role, oncotherapy.

## Abstract

Secreted frizzled-related protein 4 (sFRP4), the largest member of the secreted frizzled-related protein (sFRP) family, contains two functional domains: a cysteine-rich domain (CRD) homologous to the Wnt-binding region of frizzled (FZD) receptors and a netrin-like (NTR) domain structurally similar to axonal guidance proteins. By modulating the Wingless/Integrated (Wnt) signaling pathway, sFRP4 regulates essential cellular processes including proliferation, differentiation, apoptosis, and tissue homeostasis. This review aims to provide a comprehensive overview of the dualistic roles of sFRP4 in cancer, highlighting its tumor-suppressive and tumor-promoting functions, underlying molecular mechanisms, and therapeutic potential. A systematic literature search was conducted in PubMed and Web of Science databases (1996–2025) using predefined keywords, and from 277 identified publications, 47 studies were included that comprised clinical data, *in vitro* cell models, and *in vivo* experimental systems. Findings demonstrate that sFRP4 frequently acts as a tumor suppressor by sequestering Wnt ligands, suppressing cancer stem cell-like properties, reprogramming tumor metabolism, inhibiting angiogenesis, and enhancing chemosensitivity. Its downregulation is often driven by promoter hypermethylation or repression mediated by microRNAs (miRNAs). Conversely, in gastrointestinal and prostate cancers, sFRP4 is frequently upregulated, where it promotes Wnt pathway activation, invasion, stemness, chemoresistance, and reshaping of the tumor immune microenvironment. Mechanistic insights indicate that post-translational modifications and nuclear localization of sFRP4 further contribute to its paradoxical context-dependent functions. In conclusion, sFRP4 exerts dual roles in tumorigenesis, acting either as a tumor suppressor or promoter depending on tissue type, tumor microenvironment, and regulatory mechanisms. This complexity underscores both the challenges and opportunities of targeting sFRP4 in oncology, and future therapeutic strategies incorporating recombinant proteins, synthetic peptides, and nanoparticle-based delivery systems hold promise for harnessing its anti-tumor potential while overcoming resistance mechanisms.

## Introduction

The Secreted Frizzled-Related Protein (sFRP) family represents the largest group of Wnt inhibitors. The prototypical member of this family, known as Frizzled-related zinc-binding protein (Frzb), was initially characterized through evolutionary analysis, which demonstrated significant amino acid sequence homology with the ligand-binding domains of frizzled (FZD) transmembrane receptors [1]. FZDs are a class of transmembrane proteins within the G protein-coupled receptor (GPCR) superfamily and play a crucial role in the Wnt signaling pathway [2]. In 1997, Leyns et al. [3] established that sFRP1 functions as a Wnt antagonist. This finding was subsequently expanded upon by Melkonyan, who identified additional members of this family [4]. The sFRP family consists of five evolutionarily conserved paralogs that can be categorized into distinct phylogenetic clusters based on their genomic architecture. Phylogenetic analysis indicates that subgroup I (*SFRP1/2/5*) is encoded by tri-exonic genes located at chromosomal loci 8p12-p11.1, 4q31.3, and 10q24.1. In contrast, subgroup II (*SFRP3/4*) exhibits a multi-exonic organization, characterized by six coding exons and localized at 2q31-q33 and 7p14-p13, which correlates with alternative splicing patterns in the modulation of Wnt signaling [5].

sFRP4 is the largest member of the sFRP family and plays a crucial role in the extracellular environment by regulating biological processes, including cell signaling and proliferation. Structurally, sFRP4 features an N-terminal cysteine-rich domain (CRD) characterized by a Frizzled-like motif, alongside a C-terminal heparin-binding netrin-like (NTR) domain. The cysteine-rich CRD is closely associated with the antagonism of the Wnt signaling pathway [3]. This domain is homologous to the CRD of Frizzled, which is known to interact with Wnt proteins. The CRD of sFRP4 exhibits high sequence similarity, containing 10 conserved cysteine residues and displaying highly conserved disulfide bonds [6]. The NTR domain comprises approximately 120 amino acids and 6 cysteine residues. Beyond sFRPs, there are up to seven distinct protein families or subfamilies, such as axonal guidance factors (netrins), complement proteins C3, C4, C5, and procollagen C-endopeptidase enhancer proteins (PCOLCEs), that share homology with the NTR domain of sFRPs [7]. Notably, the NTR domain of sFRP4 possesses a lower positive charge compared to other family members, suggesting enhanced transport from the secretion site to distant target cells. As a result, sFRP4 demonstrates a weaker affinity for heparin but exhibits stronger binding to Wnt proteins via its CRD [8]. Both the CRD and NTR domains are essential for optimal Wnt inhibition [9].

sFRP4 is widely recognized as an inhibitor of the Wnt/β-catenin signaling pathway [10]. The Wnt/β-catenin cascade is an ancient and highly conserved signaling pathway present in various species, including Drosophila and mammals. The Wnt family consists of 19 identified secreted proteins in humans, which operate in autocrine or paracrine manners. The initiation of Wnt/β-catenin signaling primarily relies on Wnt1, Wnt2, Wnt3, Wnt3a, Wnt8b, Wnt10a, and Wnt10b [11]. Upon secretion, these Wnt proteins bind to FZD receptors and LRP5/6 co-receptors, thereby triggering a signaling cascade. The key downstream effector, β-catenin, evades ubiquitin-mediated degradation by avoiding phosphorylation from the cytoplasmic APC/Axin/GSK3β destruction complex. This evasion results in the accumulation of β-catenin and its subsequent translocation to the nucleus. In the nucleus, β-catenin utilizes its armadillo repeat domain to form a tripartite transcriptional activation complex with TCF/LEF transcription factors and chromatin-modifying coactivators, driving the expression of Wnt target genes [12]. Notably, sFRP4 demonstrates a general affinity for Wnt proteins [13]. Throughout this process, β-catenin acts as a central signaling mediator.

sFRP4 regulates Wnt signaling through several distinct mechanisms:

(1) sFRP4 competitively inhibits Wnt signaling via CRD- or NTR-mediated ligand sequestration, effectively blocking the formation of the Wnt-FZD receptor complex and subsequent engagement of the LRP5/6 co-receptors [14].

(2) sFRP4 may antagonize other members of the sFRP family, thereby modulating their activity [15].

(3) sFRP4 prevents Wnt from binding to FZD receptors, inhibiting downstream signal transmission [16].

(4) In certain contexts, sFRP4 can promote Wnt-FZD interactions by simultaneously binding to both molecules, thereby enhancing signal activation [17, 18].

(5) sFRP4 is involved in the extracellular transport of Wnt ligands [19, 20].

Conversely, the production and intracellular transport of sFRP4 are regulated by Wnt-mediated signaling mechanisms [21]. Given the critical role of Wnt signaling in oncogenesis, sFRP4 is generally regarded as a tumor suppressor. However, due to the complex interplay between Wnt and other signaling pathways, potential context-dependent oncogenic properties of sFRP4 have also been suggested [22].

While previous studies have primarily focused on the extracellular functions of sFRP4 as a secreted Wnt modulator, recent evidence indicates that it also exhibits concentration-dependent bidirectional roles within the nucleus. In cells with high levels of β-catenin and low levels of nuclear sFRP4, β-catenin and sFRP4 interact exclusively through their C-terminal regions, suggesting a higher binding affinity of the sFRP4 C-terminus for β-catenin. This selective interaction enhances the transcriptional activity of β-catenin. Conversely, in conditions characterized by low β-catenin and high levels of nuclear sFRP4, sFRP4 binds to β-catenin at both its N- and C-termini. Importantly, the inhibitory effect of sFRP4 binding to the N-terminus of β-catenin outweighs the promoting effect of C-terminal binding [23]. This stoichiometry-dependent switch elucidates the molecular mechanism underlying sFRP4’s context-dependent roles in tumorigenesis. Additionally, sFRP4 has demonstrated DNA-binding capacity in the nucleus. Luciferase assays and ChIP-qPCR confirmed the recruitment of sFRP4 to the promoter of Dickkopf-1 (*DKK1*), another Wnt antagonist, thereby functioning as a transcription factor that regulates *DKK1* expression [24].

**Figure 1.**
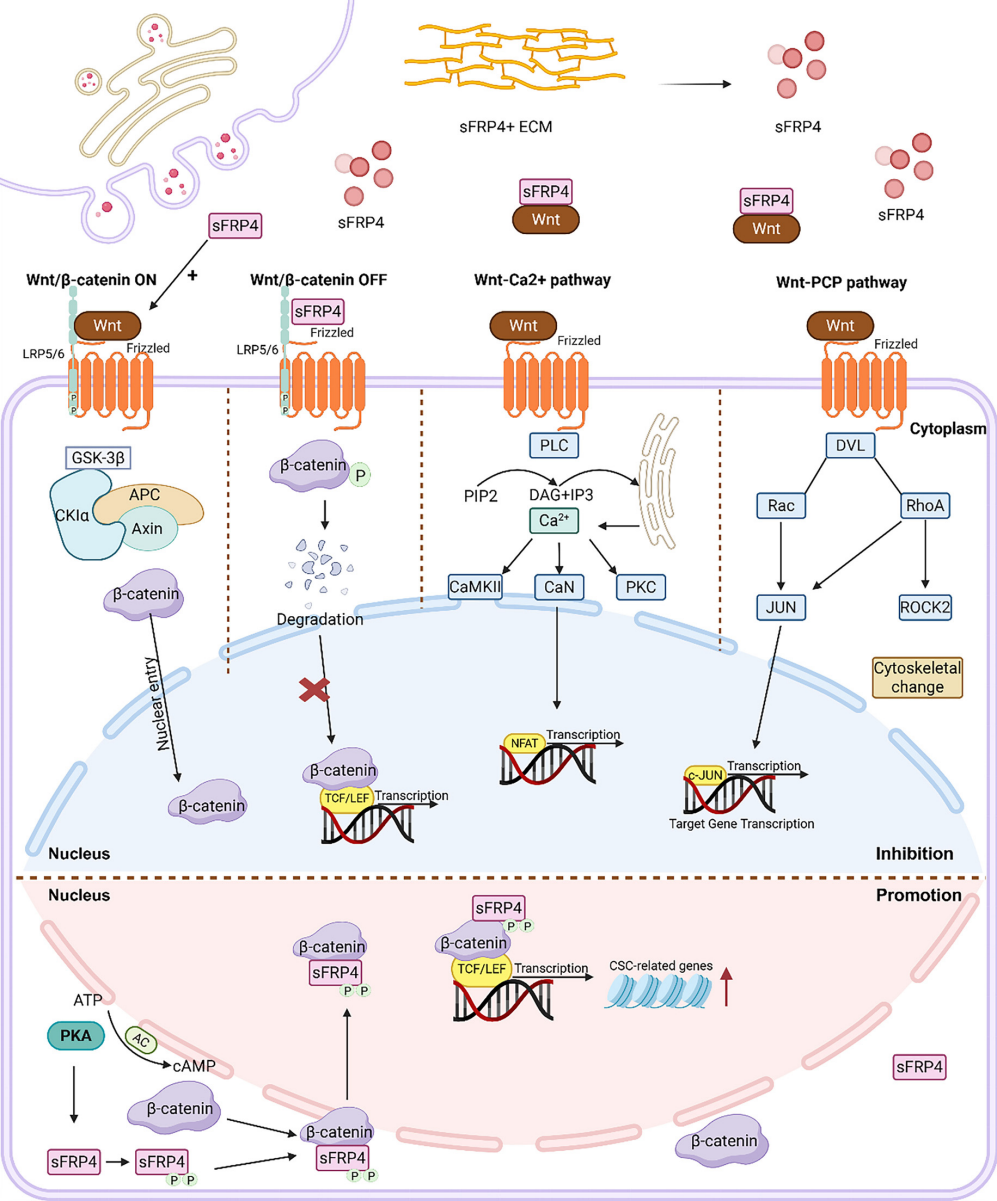
**Mechanism of sFRP4 in regulating the Wnt signaling pathway in tumors.** This diagram illustrates the main inhibitory mechanisms by which sFRP4 acts as an inhibitor of the Wnt signaling pathway. By binding to Wnt proteins through its CRD or NTR, sFRP4 sequesters Wnt ligands, preventing their interaction with downstream FZD and LRP5/6 receptors. During Wnt/PCP signaling, Wnt binds to the Fzd receptor, activating Dvl/Dsh. This in turn activates the small GTPases Rho/Rac and JNK, leading to the expression of genes related to cell polarity. In the Wnt/Ca^2+^ pathway, Wnt protein activates PLC, releasing intracellular Ca^2+^ and inhibiting the canonical Wnt signaling pathway. Under certain conditions, sFRP4 promotes the interaction between Wnt and Fzd. PKA can phosphorylate sFRP4, and the phosphorylated sFRP4 binds to β-catenin and translocates into the nucleus, where it enhances LEF/TCF transcriptional activity, leading to increased transcription of stemness-related genes. Abbreviations: APC: Adenomatous polyposis coli; ATP: Adenosine triphosphate; cAMP: Cyclic adenosine monophosphate; CaMKII: Calcium/calmodulin-dependent protein kinase II; CaN: Calcineurin; cJUN: Jun proto-oncogene; CRD: Cysteine-rich domain; CSC: Cancer stem cell; DAG: Diacylglycerol; DVL/Dsh: Dishevelled protein; ECM: Extracellular matrix; FZD/Fzd: Frizzled receptor; GSK3β: Glycogen synthase kinase 3 beta; JNK: C-Jun N-terminal kinase; LEF: Lymphoid enhancer-binding factor; LRP5/6: Low-density lipoprotein receptor-related protein 5/6; NFAT: Nuclear factor of activated T-cells; NTR: Netrin-like domain; PCP: Planar cell polarity; PIP2: Phosphatidylinositol 4,5-bisphosphate; PKA: Protein kinase A; PKC: Protein kinase C; PLC: Phospholipase C; Rac: Ras-related C3 botulinum toxin substrate; RhoA: Ras homolog family member A; ROCK2: Rho-associated coiled-coil containing protein kinase 2; sFRP4: Secreted frizzled-related protein 4; TCF: T-cell factor; Wnt: Wingless/Integrated signaling pathway.

The Wnt signaling cascade exhibits significant evolutionary conservation, functioning across a wide range of phylogenetically diverse organisms, from invertebrate models to mammals. It orchestrates various cellular activities, including mitotic regulation, lineage specification, programmed cell death, tissue homeostasis, and progenitor cell regeneration [25]. In cancer, aberrant activation of the Wnt pathway is closely linked to several malignancies, including liver and breast cancers [26]. The Wnt/β-catenin cascade plays a crucial role in regulating cancer stem cells (CSCs) [27], the metabolic reprogramming of tumor cells [28], and chemoresistance [29]. A schematic overview of these interaction is presented in Figure 1.

**Figure 2.**
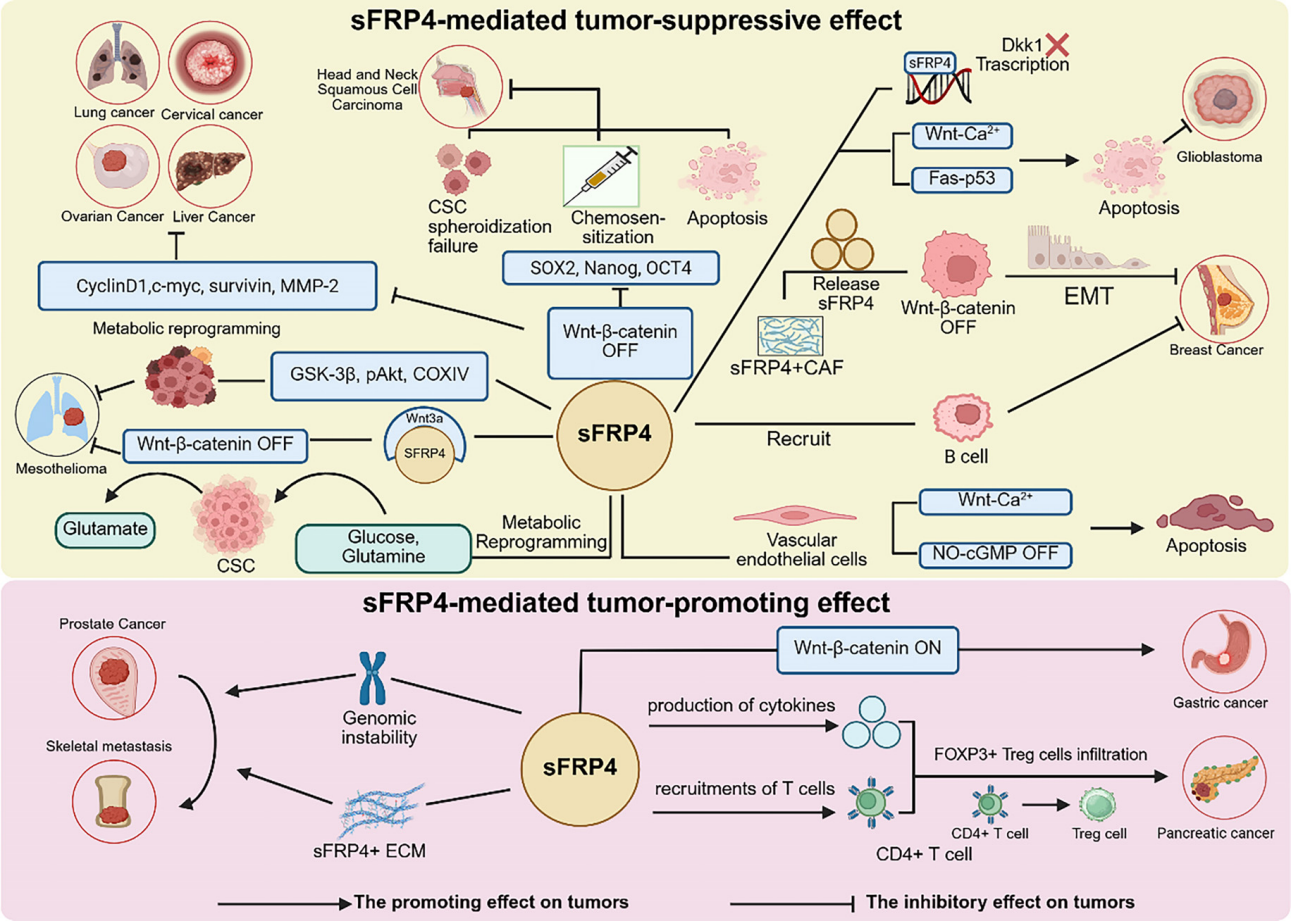
**sFRP4 plays diverse roles in different tumors.** In ovarian cancer, liver cancer, lung cancer, and cervical cancer, sFRP4 inhibits the Wnt signaling pathway, thereby suppressing the expression of Cyclin D1, c-Myc, Survivin, and MMP-2, which collectively restrain tumor progression. In head and neck squamous cell carcinoma, sFRP4 inhibits the Wnt pathway, leading to downregulation of stemness markers (SOX2, Nanog, OCT4) in cancer stem cells, resulting in impaired spheroid formation, increased apoptosis, and enhanced chemosensitivity. In breast cancer, sFRP4 recruits B cells to suppress tumor progression, and sFRP4-positive CAFs secrete sFRP4 to inhibit the Wnt pathway in tumor cells, further blocking EMT and tumor growth. In glioma, sFRP4 promotes tumor cell apoptosis by inhibiting the Wnt-Ca^2+^ and Fas-p53 pathways and acts as a transcription factor to regulate *DKK1* expression. In pleural mesothelioma, sFRP4 binds to Wnt3a to prevent Wnt pathway activation and induces metabolic reprogramming in cancer stem cells by modulating GSK-3β, pAkt, and COXIV, thereby suppressing tumor progression. Additionally, sFRP4 triggers apoptosis in vascular endothelial cells via the Wnt-Ca^2+^ and NO-cGMP pathways. sFRP4 exerts anti-proliferative effects, disrupts tumor spheroids, and reduces glucose uptake, glutamine uptake, glutamate secretion, and redox activity. However, in prostate cancer, sFRP4 expression is positively correlated with FOXP3+ Treg cell infiltration. sFRP4 promoted the secretion of T cell specific cytokines and increased the recruitment of CD4+ T cells, which may promote the Treg differentiation process. Collectively, these findings highlight sFRP4 as a novel prognostic biomarker and potential therapeutic target in pancreatic cancer. Abbreviations: CAF: Cancer-associated fibroblast; COXIV: Cytochrome c oxidase subunit IV; CSC: Cancer stem cell; Dkk1: Dickkopf-related protein 1; EMT: Epithelial-mesenchymal transition; FOXP3: Forkhead box protein P3; GSK-3β: Glycogen synthase kinase 3 beta; MMP-2: Matrix metalloproteinase-2; NO: Nitric oxide; OCT4: Octamer-binding transcription factor 4; pAkt: Phosphorylated protein kinase B; SOX2: Sex-determining region Y-box 2; Treg: Regulatory T cell; Wnt: Wingless/Integrated signaling pathway.

In addition to its functions within the Wnt signaling pathway, sFRP4 plays a crucial role in various other signaling mechanisms. It appears that sFRP4 sequesters intermediates of the PI3K/Akt pathway, which may facilitate the induction of apoptosis through mechanisms that are independent of canonical Wnt signaling [30, 31]. Furthermore, sFRP4 promotes apoptosis by activating the reactive oxygen species (ROS) pathway, which subsequently initiates the Fas-p53 pathway [32].

## Methods

To conduct a comprehensive literature review, we performed an extensive search of the Web of Science and PubMed databases using specifically tailored keywords to identify the most relevant publications. The search terms included “Secreted Frizzled-Related Protein 4” OR “sFRP4” AND “cancer” OR “neoplasm” OR “carcinoma,” covering publications from approximately 1996–2025. The initial search yielded 277 relevant articles. We then applied additional screening criteria to refine the selection, focusing specifically on studies investigating sFRP4 across various tumor types. Priority was given to research that included clinical data from human patients, *in vitro* cell line experiments, and *in vivo* animal studies to facilitate a comprehensive assessment of the role of sFRP4 in cancer. After rigorous screening, a total of 47 articles were included in the final analysis.

## Anti-tumor effects of sFRP4

sFRP4 functions as a tumor suppressor by sequestering Wnt ligands and inhibiting β-catenin activation. It exhibits significant anti-oncogenic effects across various malignancies, including hepatocellular carcinoma [33], ovarian cancer [34], glioma [35], uterine leiomyosarcoma [36], cervical cancer [37], and lung cancer [37, 38]. Clinically, sFRP4 serves as a valuable diagnostic biomarker for conditions such as hepatitis-associated hepatocellular carcinoma, ovarian cancer, and endometrial cancer [39, 40] (Figure 2, Table 1). Notably, the deletion or epigenetic silencing of *sFRP4* is associated with poor prognosis in breast cancer, highlighting its therapeutic potential [41]. Additionally, sFRP4 demonstrates a progressive loss during the malignant transformation from mucinous cystadenoma to borderline mucinous tumor, ultimately culminating in mucinous cystic carcinoma [42].

**Table 1:** The functions of sFRP4 on different types of cancers

**Function**	**Tumor type**	**Key findings**	**Ref.**
Anti-tumor	Breast cancer	(1) sFRP4+ cancer-associated fibroblasts (CAFs) secrete sFRP4 to inhibit breast cancer cell migration and epithelial-mesenchymal transition (EMT). (2) Exhibits anti-proliferative effects and induces spheroid disruption, reduces glucose uptake, glutamine uptake, glutamate secretion, and redox signatures in breast cancer-derived stem cells, while also promoting apoptosis within CSCs. (3) Enhancing chemosensitivity of breast cancer-derived stem cells.	[57, 61]
	Malignant mesothelioma	(1) Alter cancer cell metabolism. (2) Inhibits mesothelioma cell proliferation, migration, and antagonizes Wnt3a via its netrin-like domain.	[59, 80]
	Ovarian cancer	(1) sFRP4 target ovarian cancer stem cells by neutralizing the Wnt/β-catenin pathway, disrupting the interaction between β-catenin and CD24 and suppressing autophagy. (2) Enhancing chemosensitivity of ovarian cancer-derived stem cells.	[57, 84]
	Head and neck cancer	(1) Reverse EMT and restore the epithelial marker E-cadherin. (2) Disrupt spheroid formation of head and neck-derived stem cells.	[83]
	Lung cancer	(1) *In vitro* cell lines, sFRP4 inhibit the Wnt signaling pathway and downregulate the expression of proliferation-related genes. (2) sFRP4 expression is down-regulated in lung cancer cell.	[37, 38]
	Cervical cancer	*In vitro* cell lines, sFRP4 inhibit the Wnt signaling pathway and downregulate the expression of proliferation-related genes.	[37]
Pro-tumor	Pancreatic cancer	(1) High sFRP4 expression is positively correlated with FOXP3+ Treg cell infiltration, suggesting its role in shaping an immunosuppressive tumor microenvironment. (2) Mechanistically, sFRP4 promoted the secretion of T cell specific cytokines and increased the recruitment of CD4+ T cells, which may promote the Treg differentiation process.	[79]
	Gastric cancer	(1) In gastric cancer, sFRP4 is highly expressed and associated with poor prognosis. (2) sFRP4 promotes chemotherapy resistance in gastric cancer through activation of the Wnt signaling pathway.	[68–71, 75]
	Prostate cancer	(1) sFRP4 expression is increased in prostate cancer and further elevated in high-grade tumors. (2) sFRP4-positive stroma promotes bone metastasis of prostate cancer cells. (3) High sFRP4 expression is associated with genomic instability in prostate cancer.	[77, 78]

Abbreviations: CAF: Cancer-associated fibroblast; CSC: Cancer stem cell; EMT: Epithelial-mesenchymal transition; FOXP3: Forkhead box protein P3; NTR: Netrin-like domain; sFRP4: Secreted frizzled-related protein 4; Treg: Regulatory T cell; Wnt: Wingless/Integrated signaling pathway.

### The decrease expression of *sFRP4* in tumor

#### Downregulation of *sFRP4* in tumors due to methylation

Promoter hypermethylation of *sFRP* genes (*sFRP1-5*) is observed in various malignancies, including breast cancer, ovarian cancer, and cutaneous squamous cell carcinoma, resulting in transcriptional silencing [43, 44]. A systematic pooled analysis has established an epigenetic correlation between hypermethylation of the *sFRP4* promoter region and increased neoplastic risk, particularly in ovarian, colorectal, cervical squamous, and renal cell carcinomas [45]. DNA methylation, an epigenetic modification linked to gene silencing, can be reversed with the DNA methyltransferase (DNMT) inhibitor 5-Azacytidine (5-Aza). Treatment with 5-Aza has been shown to restore the expression of epigenetically silenced genes, including *sFRP4*. In CSCs, demethylation results in increased levels of sFRP4, GSK3β, and phosphorylated β-catenin, thereby confirming the methylation-dependent silencing of *sFRP4* [43]. Methyl-CpG-binding domain protein 2 (MBD2) and enhancer of zeste homolog 2 (EZH2), which are key components of the methylated MBD (DNA-binding domain) and PcG (Polycomb group) protein families, respectively, play crucial roles in epigenetic regulation. Co-silencing of MBD2 and EZH2 synergistically restored *sFRP4* expression and more effectively inhibited the proliferation of colorectal carcinoma cells [46]. Importantly, *sFRP4* promoter hypermethylation shows promise as a diagnostic biomarker in cervical squamous carcinoma [47].

#### Repression of *sFRP4* expression by microRNA (miRNA) in tumors

miRNAs are small non-coding RNAs, approximately 18–25 nucleotides in length, that regulate gene expression post-transcriptionally. They achieve this by binding to the 3’ untranslated regions (UTRs) of target mRNAs, resulting in either translational repression or mRNA degradation. miRNAs play a crucial role in cellular differentiation, development, metabolism, and the pathogenesis of diseases [48].

Elevated expression of miR-96-5p in cervical squamous cell carcinoma specimens demonstrates a stage-dependent increase and association with lymphovascular involvement. Mechanistic validation revealed *sFRP4* as a functional target of miR-96-5p, with genetic ablation of *sFRP4* reversing miR-96-5p-mediated oncogenic transformation in cervical epithelial cells [49]. Additionally, miR-181a targets *sFRP4*, thereby modulating Wnt signaling to promote stemness and platinum resistance in ovarian cancer [50]. MiR-103b directly targets *sFRP4* and has potential as a biomarker for the early diagnosis of lung adenocarcinoma [51, 52]. Additionally, MiR-31 enhances cancer cell proliferation by downregulating *sFRP4* expression in lung cancer cells. Moreover, MiR-942 promotes the stemness phenotype in esophageal squamous cell carcinoma (ESCC) by inhibiting *sFRP4* expression [53].

### sFRP4 exerts its tumor-suppressive role via the Wnt signaling pathway

#### Suppressing CSC-like properties

The Wnt signaling pathway is crucial in tumorigenesis and cancer progression. Activation of the Wnt pathway is linked to resistance against chemotherapy and radiotherapy, thereby reducing treatment efficacy [12]. The concept of “stemness” in tumor cells refers to their ability to adopt stem cell-like characteristics, including self-renewal, proliferation, differentiation, and tumorigenic potential. CSCs play a vital role in tumor development, recurrence, and drug resistance [54]. In addition to their self-renewal and migratory capabilities, CSCs utilize mechanisms to eliminate toxic substances and chemotherapeutic agents. This includes the overexpression of ATP-dependent efflux pumps, such as ABCG2, and the activation of enhanced DNA repair systems, both of which contribute to chemoresistance [55, 56].

#### Enhancing tumor chemosensitivity

In various cancers, including breast cancer, glioma, and ovarian cancer cell lines, combination therapy utilizing sFRP4 with chemotherapeutic agents effectively reduces CSC viability, diminishes sphere-forming ability, downregulates stemness-related genes, upregulates pro-apoptotic markers, and enhances chemosensitivity [57]. Notably, sFRP4 upregulation was observed in the chemotherapy-responsive A2780 cell line, while enforced expression of sFRP4 in the cisplatin-resistant A2780-Cis cell line resensitized these cells to platinum-based therapies [42]. Additionally, the activation of sFRP4 through the inhibition of miR-181a significantly decreases cisplatin resistance and stemness in high-grade serous ovarian cancer (HGSOC) [50].

#### Triggering metabolic reprogramming

The metabolic profile of tumor cells significantly differs from that of normal cells. These metabolic alterations not only facilitate rapid proliferation and survival but also provide adaptive advantages in hostile microenvironments, a characteristic feature of tumor cell metabolism known as metabolic reprogramming [58]. Compared to most tumor cells, CSCs may demonstrate elevated glycolytic activity. Research has shown that sFRP4 exerts anti-proliferative effects, induces spheroid disruption, and decreases glucose and glutamine uptake, glutamate secretion, redox signatures, and signaling cascades essential for cell survival, while concurrently promoting apoptosis within CSCs. These findings suggest that sFRP4 serves as a regulator of CSC metabolic reprogramming, potentially through the modulation of Wnt/β-catenin-dependent bioenergetic pathways [59]. In malignant mesothelioma (MM) cells treated with sFRP4 and Wnt3a, significant decreases in cytochrome c oxidase levels were observed, indicating that sFRP4 may function by suppressing cancer cell metabolism and ultimately inducing cell death [31].

#### Though cancer-associated fibroblasts (CAF) secretion to inhibit tumor progression

CAFs are a critical cell type within the tumor microenvironment (TME), playing essential roles in tumor progression. Typically, CAFs originate from normal fibroblasts that undergo phenotypic and functional transformations due to the influence of tumor cells or other signaling factors during tumor development. Among all stromal cells in the TME, CAFs are the most abundant and are closely linked to tumor progression. They secrete a variety of growth factors, cytokines, and proteins, including fibroblast growth factor (FGF) and transforming growth factor-β (TGF-β), which promote tumor cell proliferation and invasion. Additionally, CAFs release proteases, such as matrix metalloproteinases (MMPs), facilitating stromal remodeling to support tumor cell invasion and metastasis. Furthermore, CAFs regulate immune responses within the TME by suppressing immune cell activity, thereby aiding tumor immune evasion. They also participate in tumor-associated angiogenesis, promoting vascularization and nutrient supply to tumors [60].

sFRP4-expressing CAFs inhibit Wnt pathway activation in mammary carcinoma through the paracrine secretion of the sFRP4 protein, subsequently limiting tumor cell motility and obstructing molecular transitions related to epithelial-mesenchymal plasticity [61]. Furthermore, sFRP4 hinders the differentiation of adipose-derived stem cells (ADSCs) into CAFs in breast cancer, thereby slowing tumor progression [62].

### Suppressing the proliferation and migration of vascular endothelial cells

Angiogenesis is essential for tumor growth and metastasis. The neovascular network of tumors acts as a metabolic lifeline, supplying glucose and glutamine to support aerobic glycolysis while optimizing the hypoxic niche for malignant expansion. Furthermore, this network facilitates the removal of metabolic waste, contributing to the maintenance of homeostasis within the TME. These blood vessels also serve as pathways for tumor cells to enter the bloodstream, thereby promoting metastasis. Additionally, the abnormal structure of tumor vasculature can hinder immune cell infiltration, facilitating tumor immune evasion [63]. sFRP4 suppresses tumor angiogenesis by disrupting nitric oxide-cyclic guanosine monophosphate (NO-cGMP) signaling and increasing ROS levels, which leads to endothelial dysfunction [64]. Both the CRD and the NTR domain exhibit anti-angiogenic effects by elevating intracellular calcium through the Wnt-Ca^2+^ pathway via distinct mechanisms: the CRD impairs vascular network formation, while the NTR facilitates apoptosis in endothelial cells [65].

## Pro-tumorigenic effects of sFRP4

Research has shown that sFRP4 expression is upregulated in tumors derived from the gastrointestinal tract and in prostate cancer. Specifically, gastric cancer specimens display significant overexpression of sFRP4, which correlates with adverse prognostic indicators and serves as a crucial immune-related factor with important implications for immunotherapy guidance [66]. In prostate cancer, sFRP4 expression is elevated compared to normal prostate tissue, with even higher levels observed in high-grade tumors [67] (Figure 2, Table 1).

### Promoting tumor progression via the Wnt signaling pathway

Recent oncological research has revealed complex dynamics in the expression of sFRP4 and its functional implications across various cancers, highlighting inter-tumoral heterogeneity as a significant confounding variable. Notably, sFRP4 expression is markedly elevated in advanced gastric cancer [68], and shows a positive correlation with tumor invasiveness [69].

sFRP4 and Caudal Type Homeobox 1 (CDX1) have been identified as predictive biomarkers for extra-gastric recurrence following radical gastrectomy [70]. Furthermore, sFRP4 plays a significant role in gastric cancer chemoresistance; cells resistant to cisplatin or oxaliplatin display increased expression of sFRP4 and β-catenin, along with nuclear translocation of β-catenin, compared to their chemosensitive counterparts [71]. In colorectal cancer specimens, a significant downregulation of *sFRP1* and *sFRP5* mRNA was observed in 85% and 80% of cases, respectively, while *sFRP4* was overexpressed in 80% of the analyzed tumor samples. This distinctive expression pattern indicates that sFRP4 may have unique biological functions in gastrointestinal tumors, differing from those of other members of the sFRP family [72].

What factors lead to the upregulation of sFRP4 expression in gastrointestinal tumors? Research indicates that sFRP4 in colorectal tumors exhibits the lowest inhibitory activity against Wnt signaling among the sFRPs, with a methylation frequency of only 17% [73]. In gastric carcinoma, the prevalence of *sFRP4* methylation is similar in both neoplastic and paraneoplastic tissues. In contrast, hypermethylation of the *sFRP2* promoter shows a progressive detection gradient: 73.3% in carcinomas, 37.5% in premalignant intestinal metaplasia lesions, and 20% in mucosal controls [74]. These findings suggest that differential promoter methylation patterns and varying Wnt inhibitory capacities likely drive the distinct expression profiles of sFRP4 across gastrointestinal malignancies.

In addition to this mechanism, the oncogenic role of sFRP4 is further supported by its post-translational modification. Phosphorylation of sFRP4 by protein kinase A (PKA) at threonine residues T186 and T189 increases its affinity for the β-catenin/TCF4 complex, thereby enhancing Wnt signaling transcriptional activity. This PKA-dependent phosphorylation of sFRP4 transforms it from a Wnt antagonist into a potent agonist, promoting stemness and contributing to chemoresistance [75] (Figure 1).

### Increasing the invasive tumor phenotype

Studies have consistently reported elevated expression of *sFRP4* in prostate cancer cell lines (lymph node carcinoma of the prostate [LNCaP], prostate cancer-3 [PC-3/PC3], DU145, and 22Rv1) compared to control lines (PWR-1 and RWPE-1) [76]. The cytoplasmic localization of sFRP4 serves as a biomarker for poor prognostic outcomes [77]. Importantly, sFRP4 may enhance osteoblast activity and facilitate metastatic progression in prostate cancer, a mechanism similar to that of the Wnt inhibitor DKK1. Research indicates that Wnt inhibition by DKK1 decreases osteoblast differentiation and promotes an osteolytic phenotype in lesions, contributing to the aggressiveness of prostate cancer. Additionally, high expression levels of *sFRP4* in prostate cancer are strongly associated with genomic instability [78].

### Enhancing pro-tumor immunity

sFRP4 plays a significant role in modulating tumor immunity in pancreatic cancer. Research indicates that sFRP4 expression is positively correlated with the infiltration of FOXP3+ regulatory T cells (Treg). Additionally, sFRP4 enhances the secretion of T cell-specific cytokines and increases the recruitment of CD4+ T cells, which may facilitate the differentiation of Tregs. Collectively, these findings position sFRP4 as a novel prognostic biomarker and a potential therapeutic target in pancreatic cancer [79].

## Clinical targeting of sFRP4 in cancer therapy

### Recombinant sFRP4 (r-sFRP4) in preclinical models

Although the functional roles of sFRP4 in tumors are context-dependent, therapeutic strategies targeting sFRP4 have shown promising anti-tumor efficacy. Treatment of HeLa (cervical cancer) and A549 (lung cancer) cells with purified r-sFRP4 resulted in a dose-dependent inhibition of cell growth by up to 40%. Increased levels of phosphorylated β-catenin and downregulation of pro-proliferative genes (*cyclin D1*, *c-myc*, and *survivin*) indicated suppression of the Wnt signaling pathway [37]. Similarly, r-sFRP4 treatment significantly decreased cell viability and migration while enhancing adhesion in uterine leiomyosarcoma cells [36]. In MM cells, r-sFRP4 inhibits proliferation and migration, primarily through its netrin-related motif (NTR), with limited involvement from the CRD. Additionally, sFRP4 suppresses Wnt3a signaling in MM cells [80]. Treatment of serous ovarian cancer cell lines with r-sFRP4 inhibited β-catenin-dependent Wnt signaling and reduced transcription of Wnt target genes (*Axin2*, *Cyclin D1*, and *Myc*). This treatment also enhanced cell adhesion, decreased migration, and promoted a shift toward an epithelial phenotype, characterized by upregulation of E-cadherin and downregulation of mesenchymal markers (*Vimentin* and *Twist*) [81]. Furthermore, sFRP4 exhibits anti-proliferative activity against CSCs derived from breast, prostate, ovarian, glioblastoma, and head and neck tumors, while simultaneously enhancing chemosensitivity [57, 82, 83]. These findings suggest that combining chemotherapy with sFRP4 may improve outcomes in conventional cancer treatments.

### CRD and NTR-derived micropeptides

Synthetic micropeptides targeting the CRD and the NTR of sFRP4 significantly reduced CSC marker expression, inhibited angiogenesis, upregulated pro-apoptotic genes, and enhanced the sensitivity of ovarian CSCs to cisplatin. These synthetic polypeptides functioned through a dual mechanism of action, attenuating the canonical Wnt/β-catenin signaling pathway while simultaneously disrupting the β-catenin-CD24 molecular interactions. Additionally, they effectively inhibited autophagy, a critical survival mechanism for CSCs [84]. Moreover, the overexpression of isolated CRD and NTR domains in glioma cells led to a downregulation of characteristic CSC traits. Notably, the NTR domain exhibited stronger inhibitory effects than the CRD domain on MMP-2-mediated invasion and disrupted fibronectin assembly, thereby reducing cell adhesion in the LN229 glioma cell line [85].

### Other therapies

To investigate targeted delivery, CS-DS nanoparticles encapsulating the sFRP4-GFP protein were administered to multiple myeloma (MM) cells. Both sFRP4 and NTR nanoparticles significantly decreased MM cell viability, with the NTR domain exhibiting the most pronounced anti-tumor effects compared to CRD nanoparticles [86]. In a separate study, alginate-encapsulated Wharton’s jelly-derived mesenchymal stem cells (WJMSCs) were co-cultured with breast CSCs within a three-dimensional (3D) microenvironment. This 3D co-culture system, in contrast to two-dimensional (2D) models, upregulated sFRP4 expression, inhibited the Wnt pathway, and downregulated the expression of drug transporters, epithelial-mesenchymal transition (EMT)-related markers, and angiogenesis-associated genes [87].

## Conclusions and future perspectives

sFRP4 is widely recognized as an inhibitor of the canonical Wnt signaling pathway. However, recent evidence has revealed its paradoxical dual roles in cancer: it can act as a tumor suppressor, inhibiting tumor progression, while also promoting cancer in gastrointestinal tumors. sFRP4 exerts its effects through the Wnt and PI3K/Akt signaling pathways. As an emerging therapeutic target in oncology, treatments that focus on genes with oncogenic alterations and associated signaling pathways are expected to remain a crucial cancer treatment modality, given that cancer is fundamentally a genetic disease driven by such alterations [88]. sFRP4 plays a pivotal role in regulating tumor growth, invasion, and the immune microenvironment. Despite significant advancements in research, challenges such as drug resistance and targeting specificity persist. Future sFRP4-targeted therapies, supported by multi-target combination strategies, precision medicine, and innovative technologies, hold promise for delivering more effective treatment regimens to cancer patients, ultimately enhancing survival outcomes and quality of life.
